# Psychiatric care following deliberate self-harm and prospective mortality: evidence from a national cohort of patients in routine care

**DOI:** 10.1192/j.eurpsy.2022.336

**Published:** 2022-09-01

**Authors:** P. Qin

**Affiliations:** University of Oslo, National Centre For Suicide Research And Prevention, Oslo, Norway

**Keywords:** deliberate self-harm, prospective outcome, mental healthcare, treatment engagement

## Abstract

**Introduction:**

Psychiatric care following self-harm treatment is pivotal in patients´ life both in short- and long-terms.

**Objectives:**

To examine follow-up psychiatric care received by patients treated for deliberate self-harm (DSH), and to assess the influence of psychiatric referral and treatment attendance on risk for subsequent mortality.

**Methods:**

Nationwide registries were interlinked to follow all DSH patients for data on personal socioeconomic status, clinical features of DSH, psychiatric treatment and cause of death. Data were analyzed with Logistic regression and cause-specific survival analysis.

**Results:**

The study identified 43153 patients involving 69569 DSH episodes. Of these patients 15.7% were referred or transferred to psychiatric services and 51.0% attended psychiatric treatment within subsequent 3 months. Evidently, prior psychiatric history and psychiatric comorbidities had strong influence on both referral and attendance to psychiatric healthcare, personal socioeconomic status also deviated the likelihoods. During the follow-up, 7041 patients died from suicide (n=911) or other causes (n=6130). While suicide risk was highly associated with male gender, middle age, and particularly, prior and coexisting psychopathologies, other cause mortality was strongly associated with old age and socioeconomic disadvantages. Meanwhile, a referral to psychiatric services was associated with suicide risk, and the risk was particularly high for patients who received the referral but did not attend psychiatric treatment. The observed effect was more pronounced during the early years, and in patients of young or middle age and those with a clear intent of self-harm.

**Conclusions:**

The insightful findings highlight the importance of patients´ attendance and engagement in follow-up psychiatric care on risk for subsequent mortality.

**Disclosure:**

No significant relationships.

